# Kinetic and Methodological Insights into Hydrophilic Drug Release from Mesoporous Silica Nanocarriers

**DOI:** 10.3390/pharmaceutics17060694

**Published:** 2025-05-25

**Authors:** Rodrigo Rozas, Andrea C. Ortiz, Sofía Peñaloza, Sebastián Lizama, Mario E. Flores, Javier Morales, Francisco Arriagada

**Affiliations:** 1 Departamento de Ciencias y Tecnología Farmacéutica, Facultad de Ciencias Químicas y Farmacéuticas, Universidad de Chile, Santiago 8380494, Chile; rodrigo.rozas@alumnos.uach.cl (R.R.); sofia.penaloza@ug.uchile.cl (S.P.); 2 Instituto de Farmacia, Facultad de Ciencias, Universidad Austral de Chile, Valdivia 5090000, Chile; slizama@alumnos.uach.cl; 3 Escuela de Química y Farmacia, Facultad de Ciencias, Universidad San Sebastián, Santiago 7510157, Chile; andrea.ortizo@uss.cl; 4 Instituto de Ciencias Químicas, Facultad de Ciencias, Universidad Austral de Chile, Valdivia 5090000, Chile; mario.flores@uach.cl

**Keywords:** release testing, drug release, mesoporous silica nanoparticles, in vitro methods, rhodamine B, release study

## Abstract

**Background/Objectives:** The absence of standardized protocols for assessing in vitro drug release from nanocarriers poses significant challenges in nanoformulation development. This study evaluated three in vitro methods: sample and separate without medium replacement (independent batch), sample and separate with medium replacement, and a dialysis bag method, to characterize the release of rhodamine B from mesoporous silica nanoparticles (MSNs). **Methods:** Each method was examined under varying agitation conditions (shaking versus stirring). MSNs were synthesized via the sol-gel method, exhibiting a hydrodynamic diameter of 202 nm, a zeta potential of −23.5 mV, and a surface area of 688 m^2^/g, with a drug loading efficiency of 32.4%. **Results:** Release profiles revealed that the independent batch method exhibited a rapid initial burst followed by a plateau after 4 h, attributed to surface saturation effects. Conversely, the sample and separate with medium replacement method sustained the release up to 60% over 48 h, maintaining sink conditions. The dialysis method showed agitation-dependent variability, with magnetic stirring using a longer stir bar enhancing release. Kinetic analyses indicated first-order kinetics with non-Fickian diffusion. **Conclusions:** Overall, the results indicate that both the selection of the in vitro method and the agitation technique play a crucial role in determining the apparent drug release kinetics from nanocarriers. These findings highlight the critical role of experimental design in interpreting nanocarrier release kinetics, advocating for tailored protocols to improve reproducibility and in vitro–in vivo correlations in nanoformulation.

## 1. Introduction

Standardized methods and validated protocols for regulatory approval of conventional pharmaceuticals, such as capsules and tablets, have been established in several pharmacopeias (compendial methods) or guidance documents for industry from different regulatory agencies [1,2]. Among the different methods, drug release testing protocols play a critical role in ensuring quality and safety as well as an approach to the potential efficacy of pharmaceutical dosage forms. These protocols provide detailed guidelines on the apparatus, release media, specific conditions, and sampling intervals required to ensure reproducibility and reliability [3]. In contrast, novel colloidal carriers, such as nanoformulations, lack standardized protocols for assessing in vitro drug release from their matrices, primarily due to the highly variable physicochemical properties unique to each formulation and their specific behaviors in different release media [4,5]. These properties necessitate specialized techniques or equipment that have not been validated or included in compendial methods [6]. As a result, nanoparticle drug release testing still faces significant gaps for different types of nanostructures, potentially leading to discrepancies between in vitro and in vivo performance, unpredictable therapeutic outcomes, and an increased risk of clinical trial failure [7,8]. Therefore, it is essential to comprehensively explore the impact of different drug release testing methods on the factors influencing the release kinetics of nanocarriers.

Among various nanocarriers reported, mesoporous silica nanoparticles (MSNs)-based drug delivery systems (DDSs) have emerged as promising carriers in the drug delivery field due to their high surface area-to-volume ratio, tunable pore size, and versatile surface functionalization [9]. These characteristics allow controlled drug release of a wide variety of therapeutic agents, highlighting their potential application in targeted drug delivery. Drug release testing from MSNs has been conducted using non-compendial methods, with the most popular being the sample and separate and dialysis bag techniques [10]. The sample and separate method involves dispersing drug-loaded MSNs in a release medium and withdrawing aliquots at different time intervals, with the nanoparticle separation method (e.g., centrifugation) and agitation technique (e.g., stirring or shaking) being potential sources of error [11]. In the dialysis bag method, drug-loaded MSNs are dispersed and placed inside a bag, which is then immersed in a large volume of release medium, forming inner and outer compartments. Thus, the membrane provides a physical separation of the nanoparticles from the released drug, which diffuses into the surrounding medium where aliquots can be withdrawn for direct quantification [12]. Sources of error in this method include the specific molecular weight cut-off (MWCO) of the membrane, drug-membrane interactions, inner-outer compartment ratio, and equilibrium between drug concentrations in the inner and outer compartments. Various authors have reported limitations in centrifugation-based techniques, such as challenges in fully separating drug-loaded particles from free drugs and the perturbation in interfacial equilibria caused by potential forced release induced during centrifugation [13,14,15]. Likewise, membrane-based methods have shown inherent drawbacks [12,16,17], which can lead to misinterpretation as sustained release profiles [18,19]. Furthermore, other authors have found difficulties in evaluating the interchangeability of certain testing methodologies [20].

The FDA has emphasized that a validated analytical procedure is required to assess in vitro release, using an appropriate medium and suitable agitation [21]. However, the choice of one method over another may depend on the formulation or its intended purpose. Meanwhile, the Nanotechnology Characterization Laboratory (NCL) has reported an improved ultrafiltration method [22]. By employing a stable-isotope tracer to more accurately distinguish between encapsulated and unencapsulated drug fractions, this approach enhances the evaluation of drug stability and release in biological matrices such as plasma. Recently, a bio-predictive in vitro release assay has been described, which integrates pharmacokinetic data deconvolution with a design-of-experiments framework [23].

Despite various advances, technical difficulties inherent in each method persist in routine performance testing. The lack of clear guidelines, need for adjustments in experimental conditions on a case-by-case basis, and absence of universally accepted protocols hinder the ability to compare results between different kinetic studies using MSNs and complicate their interpretation [24]. Comparative studies on the release kinetics of different types of molecules from MSNs have been widely conducted, but unlike lipid or polymeric carriers [25,26], the comparison of different release testing methods for mesoporous silica has been less frequently reported. In this study, we compared three different in vitro drug release assay methods for mesoporous silica nanoparticles (MSNs) using rhodamine B as a hydrophilic model drug. The evaluated methods included: (1) sample and separate without medium renewal (independent batches), (2) sample and separate with medium renewal, and (3) the dialysis bag method, where the agitation technique was varied between shaking and magnetic stirring for all methods. Furthermore, mathematical models were employed to characterize the release profiles, with the aim of elucidating the critical factors influencing the variability of the kinetic results based on the different techniques used.

## 2. Materials and Methods

### 2.1. Materials

Rhodamine B (RhB, CAS: 81-88-9), tetraethyl orthosilicate (TEOS, 98%), 4Å molecular sieves, hydrochloric acid fuming (HCl, acs reagent 37%), hexadecyltrimethylammonium bromide (CTAB, ≥97%), sodium hydroxide (NaOH, for analysis), and ethanol (absolute for analysis) were obtained from Merck (KGaA, Darmstadt, Germany). The SnakeSkin^TM^ dialysis tubing (10,000 MWCO) was obtained from Thermo Scientific (ThermoFisher Scientific Carlsbad, CA, USA). All other reagents and solvents were of analytical grade. Deionized water (Milli-Q, 18.2 MΩ.cm) was used in all experiments.

### 2.2. Preparation of Mesoporous Silica Nanoparticles (MSNs)

MSNs were prepared by the sol-gel technique following protocols reported elsewhere [27]. Briefly, 0.25 g of CTAB was added to 120 mL of water, followed by the addition of 0.9 mL of NaOH (2.0 M) to the mixture, which was stirred at 80 °C. After 45 min, 1.3 mL of TEOS was added dropwise and the mixture was stirred for 2 h at 80 °C. Nanoparticles were collected by centrifugation at 13,000 rpm for 20 min and washed with ethanol and water several times. The CTAB was removed through a solvent extraction method using a 0.5 M HCl/EtOH solution, refluxed at 120 °C for 6 h. The nanoparticles were thoroughly washed with ethanol and water using centrifugation cycles, and the extraction procedure was repeated twice. The final product was dried under vacuum for further experiments. Residual CTAB traces were reduced to negligible levels, monitored by FT–IR analysis.

### 2.3. Characterization

The physicochemical characterization of mesoporous silica nanoparticles was conducted by measuring their hydrodynamic diameter using dynamic light scattering (DLS) at 25 °C with a detection angle of 173° and an equilibration time of 120 s, and their zeta potential using a Malvern Zetasizer Nano ZS90 (Malvern, UK). Samples were diluted in water at 25 °C and the measurement was performed three times. Morphology and porous structure were studied using transmission electron microscopy (TEM) on a Hitachi HT7700 (Hitachi High-Technologies Corporation, Tokyo, Japan) model microscope with an accelerating voltage of 120.00 kV. Samples were prepared by dripping the suspension of nanoparticles in dry ethanol onto a Formvar/carbon-supported copper grid (300 mesh) and allowing the sample to dry before measurement. Textural properties were determined by the Brunauer–Emmett–Teller (BET) and Barrett–Joyner–Halenda (BJH) methods. The N_2_ adsorption/desorption isotherms were measured at −196 °C on a Micromeritics 3-Flex instrument (Micromeritics Instrument Corporation, Norcross, GA, USA). Fourier-transform infrared (FT–IR) spectra were recorded using a Nicolet iS5 instrument (Nicolet Instrument, Thermo Fisher Scientific, Waltham, MA, USA) with 4 cm^−1^ resolution in the range of 4000–600 cm^−1^, averaging 16 scans. UV–Vis absorption spectra were recorded on a single-beam UV–Vis Agilent 8453 spectrophotometer (Agilent Technologies, Shanghai, China) equipped with 1 cm quartz cells.

### 2.4. Drug Loading

MSNs were loaded with RhB using the impregnation method. To this, 5 mg of RhB was added to 1 mL of an aqueous suspension of nanoparticles (10 mg/mL) and the mixture was sonicated for 20 min, followed by gentle shaking at room temperature for 24 h. RhB-loaded mesoporous silica nanoparticles (MSNs@RhB) were collected by centrifugation and the supernatant was quantified using a UV–Vis calibration curve of RhB. The drug loading efficiency (%LE) and drug loading content (%LC) were calculated according to the following equations:

(1) $$\%LE=\frac{{RhB}_{in}-{RhB}_{sup}}{{RhB}_{in}}\times 100$$

(2) $$\%LC=\frac{{RhB}_{ent}}{MSNs@RhB}\times 100$$

where RhB_in_, RhB_sup_, and RhB_ent_ are the mass of rhodamine B initially used in the impregnation batch, the mass of rhodamine B in the supernatant after loading process, and the mass of rhodamine B entrapped in the nanoparticles (i.e., the mathematical difference between RhB_in_ and RhB_sup_), respectively. On the other hand, MSNs@RhB is the mass of the final nanosystem obtained after the loading process [28].

### 2.5. In Vitro Drug Release

To study the effects of different release testing methods on the kinetic profiles, the widely used PBS (10 mM) at pH 7.4 and 37 °C was selected as a standard release medium. The release studies were conducted using the dialysis method and the sample and separate method, both under shaking and magnetic stirring. For each method, the study was performed at three agitation speeds: 50, 100, and 200 rpm. Samples were taken at different time points, and fresh medium was added to maintain sink conditions. In the dialysis method, the release medium withdrawn was replenished, while in the sample and separate method with medium renewal, samples were centrifuged and the nanoparticle pellet was resuspended in fresh medium and returned to the experimental flask. Additionally, the release experiment was also conducted using the independent batch technique, where a separate batch of drug-loaded nanoparticles was used for each sampling point and left for its designated time. In this method, no medium replacement occurs; however, the volumes used are sufficient to maintain conditions that prevent drug precipitation. After each experiment concluded, an infinite point was obtained to estimate the total drug content, which was compared to the theoretical total amount of RhB. The amount of RhB in the supernatant was quantified by UV–Vis at 554 nm. The details of each methodology are described in the following sections.

#### 2.5.1. Sample and Separate with Shaking/Stirring

MSNs@RhB were dispersed in 20 mL of the release medium and maintained at 37 °C with continuous shaking in an orbital shaker or stirring with a magnetic stirrer. Samples were withdrawn at 0.15, 0.5, 1, 2, 3, 4, 6, 8, 24, and 48 h, then centrifuged (15,000 rpm for 5 min) and the supernatant was collected for analysis.

#### 2.5.2. Sample and Separate Using Independent Batch Shaking/Stirring

For each time point, a separate vial containing 20 mL of MSNs@RhB suspension was used and subjected to either shaking or stirring for 0.15, 0.5, 1, 2, 3, 4, 6, 8, 24, and 48 h. After shaking or stirring, the samples were centrifuged (15,000 rpm for 5 min) and the supernatant was collected for further analysis. A total of ten vials were used, with each time point conducted in triplicate.

#### 2.5.3. Dialysis Bag with Shaking/Stirring

The dialysis bag was briefly soaked in the release medium according to the manufacturer’s recommendations (<5 s), sealed at the bottom and filled with 1 mL of MSNs@RhB suspension. Then, the bag was sealed at the top and placed in 19 mL of release medium to reach a total volume of 20 mL. Samples were withdrawn at 0.15, 0.5, 1, 2, 3, 4, 6, 8, 24, and 48 h, with the same volume replaced by fresh medium each time. Subsequently, the samples were analysis by UV–Vis.

### 2.6. Kinetics Analysis

Release profiles were performed based on the mean ± standard deviation of at least three independent experiments and the data were expressed as cumulative percentage of RhB released versus time (h). The cumulative fraction released was determined as the ratio of the cumulative amount of drug in solution at time t relative to the amount at the infinite point. This experimental infinite point correlated well with the theoretical total amount of drug entrapped in the nanoparticles and available for release. The data were normalized to represent the percentage of drug released (F) from 0% to 100%. In this fashion, the kinetics data were fitted to mathematical models, including zero-order (Equation (3)), first-order (Equation (4)), and the power law (Equation (5)):

(3) $$F=k_{0}t$$

(4) $$F=100(1-e^{-k_{1}t})$$

(5) $$F=K_{KP}t^{n}$$

where k_0_, k_1_, and K_KP_ are the zero-order, first-order, and Korsmeyer–Peppas release apparent rate constants, respectively. The term *n* is the diffusional exponent, analyzed assuming a spherical material. Specifically, if *n* = 0.43, the mechanism corresponds to Fickian diffusion. When 0.43 < *n* < 0.85, it indicates anomalous transport (non-Fickian diffusion), while *n*~0.85 suggests Case-II transport [29].

The similarity factor (f_2_) and the difference factor (f_1_), two model-independent methods recognized by several regulatory agencies such as the FDA or EMA, were used to compare the release profiles, as shown in Equation (6) and Equation (7), respectively.

(6) $$f_{2}=50\log\left\{{\left[1+\frac{1}{n}\sum_{i=1}^{n}{\left(\mu_{1i}-\mu_{2i}\right)}^{2}\right]}^{-0.5}\cdot 100\right\}$$

(7) $$f_{1}=\left[\frac{\sum_{i=1}^{n}\left|\mu_{1i}-\mu_{2i}\right|}{\sum_{i=1}^{n}\mu_{1i}}\right]\cdot 100$$

where *n* is the number of time points while µ_1i_ and µ_2i_ represent the percentage values at each corresponding time point in a release profile 1 and the profile 2 being compared, respectively. A higher f_2_ value and a lower f_1_ value indicate greater similarity between the two profiles. Specifically, the profiles are considered similar when f_2_ falls between 50 and 100 and f_1_ is between 0 and 15 [30,31].

### 2.7. Statistical Analysis

All results are presented as mean ± SD of at least three independent experiments. The GraphPad Prism software version 8.0.1 (San Diego, CA, USA) and the DDSolver^®^ add-In (Microsoft Excel) program version 1.0 were used. The coefficient of determination (R^2^), Akaike Information Criterion (AIC), root mean square error (RMSE), and one-way ANOVA test with Tukey’s post-test were used where applicable.

## 3. Results and Discussion

### 3.1. Synthesis and Characterization of Silica Nanomaterials

**Figure 1:**
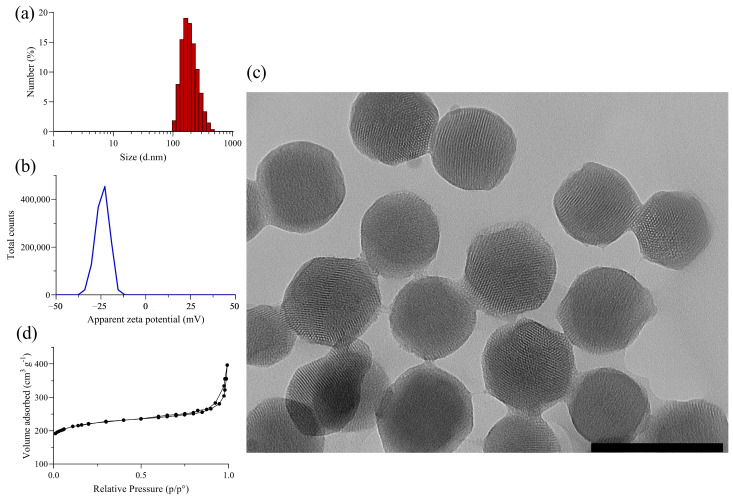
Physicochemical and textural characterization of MSN samples. (**a**) Histogram of hydrodynamic diameter distribution of MSN, (**b**) zeta potential distribution of MSN, (**c**) TEM image of MSN (scale bar: 200 nm), and (**d**) N_2_ adsorption/desorption isotherm of MSN.

Nine batches of MSNs were synthesized by the sol-gel method and fully characterized. Appropriate amounts of each stock were then used to load RhB using the impregnation method. Bare nanoparticles have an average hydrodynamic diameter (expressed as a number) of ca. 202 nm with an average peak of ~173 nm and a zeta potential of −23.5 mV, evidencing the silica nature conferred mainly by the deprotonated silanol groups (Figure 1a,b). The narrow size distribution was evidenced by a PdI value of 0.049 and the size, spherical morphology, and porous nature were corroborated by TEM images (Figure 1c). The N_2_ adsorption/desorption isotherm exhibited a type IV isotherm, confirming the typical behavior for mesoporous materials (Figure 1d). The textural properties such as surface area, pore size, and pore volume were estimated as 688 m^2^g^−1^, 3.2 nm, and 0.54 cm^3^g^−1^, respectively.

**Figure 2:**
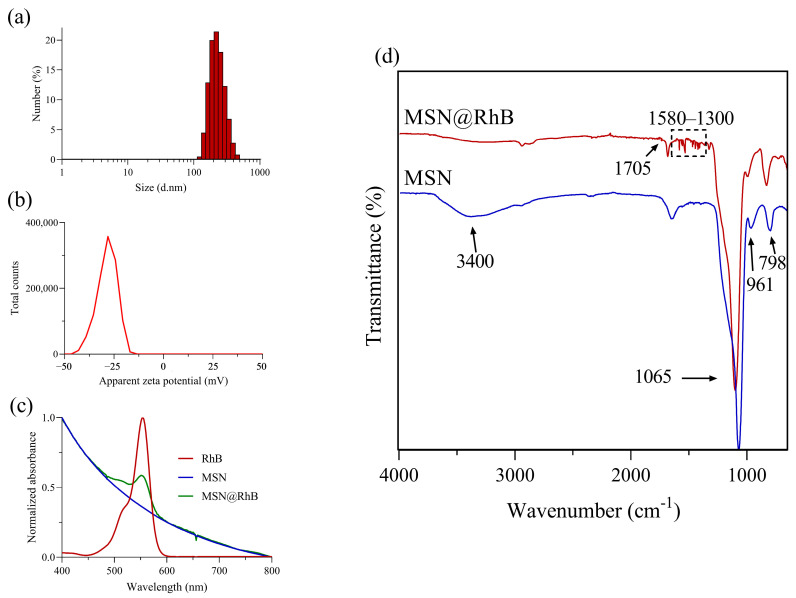
Characterization of RhB entrapped in MSN samples. (**a**) Histogram of hydrodynamic diameter distribution of MSN@RhB, (**b**) zeta potential distribution of MSN@RhB, (**c**) absorption spectra of free RhB (red curve), MSN (blue curve) and MSN@RhB (green curve), and (**d**) FTIR spectra of MSN (blue line) and MSN@RhB (red line).

After the RhB loading and purification processes, the %LE and %LC obtained were 32.4% and 13.9%, respectively. These values mean that ~338 µmol of RhB were entrapped per gram of nanoparticles. At the working pH of the loading process (7.4), there are two main interactions between nanoparticles and RhB: (i) electrostatic and (ii) hydrogen bonding. RhB is a cationic dye with a pKa value of ~4.2 [32,33]. Therefore, at pH 7.4 a zwitterion exists in which electrostatic interaction between the negative surface of the nanoparticle (Si-O^–^) and the positively charged group in RhB (-C=N^+^) is promoted. An H-bonding formation between the (-COO^–^) group in RhB and the coexisting silanol groups in the nanoparticle (Si-OH) could also be produced. Repulsive forces might also be produced between the (-COO^–^) and (-Si-O^–^), which explains the relatively low %LE. Other authors have reported the RhB adsorption on silica materials as 820 µmol/g. We attribute this difference mainly to the adsorbent materials’ characteristics, because our textural properties such as surface area and pore volume were lower [34]. Additionally, after the loading process, the hydrodynamic diameter showed a slight increase to 233 nm (Figure 2a) and the average peak reached 210 nm. These results are in agreement with the slight increase in the PdI to 0.14. On the other hand, the zeta potential changed from −23.5 to −29.0 mV (Figure 2b). Even though the change was not substantial, it could be explained by the presence of the carboxyl group in RhB.

Furthermore, that the RhB was entrapped in the MSNs was evidenced in the UV–Vis spectrum through its feature absorption band at 554 nm, contrary to bare MSNs, which only exhibited the characteristic scattering (Figure 2c). Finally, FT–IR analysis confirmed the presence of RhB on the nanoparticles (Figure 2d). A typical intense peak at 1065 cm^−1^, a broad band centered at ~3400 cm^−1^, and peaks at 961 cm^−1^ and 798 cm^−1^ characterized the siloxane asymmetric vibration, the adsorbed water and overlapped Si-OH stretching vibration, the Si-O bending, and the Si-O symmetric stretching, respectively, which are typical features of silica nanoparticles. The MSN@RhB sample exhibited characteristic peaks of RhB in the region of 1300 cm^−1^ to 1580 cm^−1^, which can be attributed to C-N, C-H, and C=C vibrations in aromatic rings and heterocycle present in the molecule [35,36]. Also, a new band, although weak, appears at 1705 cm^−1^, which could be assigned to the C=O stretching vibration, indicating the presence of the carboxylic acid of rhodamine B. Overall, the results show that mesoporous silica nanoparticles, subsequently loaded with RhB, were successfully obtained. These characteristics describe a porous material that can host RhB both on its surface and deep within its pores. Its physicochemical profile suggests good colloidal stability, a narrow (monodisperse) size distribution, and other favorable characteristics, making it ideal for drug loading and release studies. Consequently, we expect these features to enhance the quality and consistency of release experiments. In particular, since the silica matrix is not functionalized, this will allow RhB to diffuse freely, as its release is governed only by dissolution, ionization, and the electrostatic interactions discussed earlier. That simplicity makes it much more likely that differences in the observed release profiles are due to variations in the testing method rather than changes in how RhB interacts with the nanoparticle surface.

### 3.2. Release Kinetics of RhB from Mesoporous Silica Nanoparticles

#### 3.2.1. Comparison of Release Profiles Obtained by Different Methods

Considering that various experimental factors influence the release profile [37,38], specific recommendations have been provided for different pharmaceutical formulations [39]. In this context, it is well established that agitation speed affects the amount of drug released [40,41,42]. In the present work, the release of RhB from nanoparticles was studied using two main types of agitation, namely magnetic stirring and shaking. To explore the agitation speed, we performed release tests at 50, 100, and 200 rpm, and observed the same pattern (Figure S1) that others have reported: as the agitation speed increases, the percentage of the drug in the solution increases [43,44,45]. Based on our findings and the literature (which shows that 100 rpm has sufficient discriminatory capacity for these assays), we chose 100 rpm to detail the release profiles and carry out our kinetic analyses.

**Figure 3:**
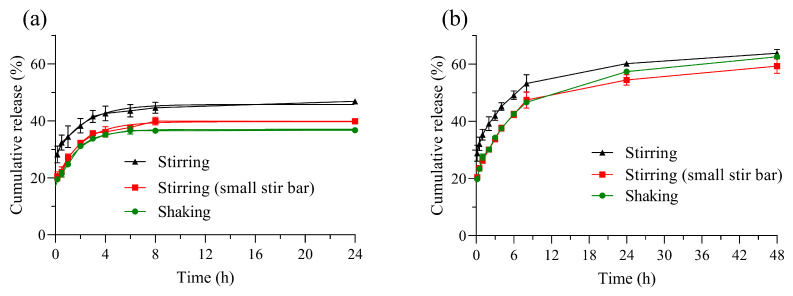
Release profile obtained with the sample and separate method. (**a**) Independent batch method, and (**b**) sample and separate with medium replacement. Stirring was performed with a 30 mm magnetic bar and the small stir bar was 20 mm long.

In addition, the size of the magnetic stir bar, and particularly the ratio between its length and the vessel diameter, may influence the results. Therefore, the stir bar length-to-vessel diameter ratio was varied by changing the stir bar size from 30 mm to 20 mm. The results indicate that a larger stir bar leads to a higher percentage of RhB release (Figure 3). According to the official dimensions for USP Apparatus II, the paddle length (74 mm) and the diameter of a 1 L nominal volume vessel (100 mm) provide a paddle-to-diameter ratio of 0.74. In this study, the experiments were conducted with a stir bar-to-diameter ratio of 0.86, while reducing the stir bar size decreased the ratio to 0.57. This factor was assessed for all methods, both with and without renewal of the release medium. In both cases, a higher ratio resulted in greater drug release. This observation can be attributed to differences in fluid velocity induced by different impellers [46,47,48], including magnetic stir bars. From a hydrodynamic perspective, the Reynolds number [49] for the smaller stir bar was estimated to be ~957, whereas for the larger stir bar, it was ~2153, which may contribute to a more efficient renewal of the diffusion layer and, consequently, a higher percentage of rhodamine B release (see the details of the calculation in Equation (S1)).

The release profiles of the samples obtained using the sample and separate method, both with independent batches and with medium replacement, exhibited an initial burst release of approximately 20%. This initial burst is mainly attributed to the weakly adsorbed dye on the nanoparticle surface, after which RhB diffuses first from near-surface pores and then from deeper pore regions, driving the release profile into a pseudo-steady state (Figure 3). For the independent batch technique (without medium renewal), a plateau was reached after ~4 h of release, regardless of whether magnetic stirring or orbital shaking was used (Figure 3a). In the case of the sample and separate method with medium renewal, the data during the first 3 h of the assay were comparable to those obtained with the independent batch method, whether under stirring or shaking conditions (Figure 3b).

However, after 3 h, the results begin to diverge, showing statistically significant differences (* *p* < 0.05) between sampling points from the 4h mark onward. These findings can be explained by considering the influence of sink conditions and the formation of a rhodamine B layer near the nanoparticle’s surface. Sink conditions refer to the principle analogous to drug removal from biological fluids, traditionally described for the gastrointestinal tract at the absorption site [50]. Under these conditions, the drug does not accumulate but rather “disappears” from the absorption site due to permeation and diffusion processes. However, a temporary nonsink condition may be an appropriate analog under certain biological circumstances [51,52]. For quality control purposes in early development of pharmaceutical products, dissolution rate evaluation methods, as recommended by various pharmacopeias, are typically conducted under sink conditions. In practice, this is achieved by ensuring experimental conditions such that the maximum drug concentration in the bulk fluid does not exceed approximately 20% of the drug’s solubility [53] or by maintaining a medium volume at least three times the required amount to form a saturated drug solution [19]. To meet this requirement in “closed system” methodologies, such as most compendial and non-compendial methods, a large volume of release medium must be used or frequent medium renewal must be performed. Nevertheless, it is known that ensuring sink conditions in the surrounding bulk fluid does not guarantee the absence of a potential drug saturation effect, even for highly soluble compounds [53,54]. In our study, results suggest that a superficial drug layer formed over time (after 4 h in our case), slowing the release process when equilibrium was reached at the surface. This phenomenon was particularly evident in the independent batch method, where no medium renewal occurs (Figure 3a). The research groups of Bergström [55] and DeShong [56] separately reported a biphasic release profile for the cationic dye rhodamine 6G, attributed to dye release from both the nanoparticle pores and the layer formed near the particle surface. These findings highlight the importance of renewing the medium at each sampling point, as even under sink conditions, the presence of a drug layer near the nanoparticle surface and interfacial adsorption/desorption phenomena may impact drug release, causing a reduction in the release rate. Consistently, other studies have emphasized the necessity of frequent medium renewal [15]. This consideration is crucial for appropriately designing experimental protocols, as it significantly affects data interpretation. Conversely, when fresh medium was replenished at each sampling point, the concentration gradient was maintained and drug release continued, reaching ~60% RhB release after 48 h (Figure 3b).

After identifying differences between the sample and separate methods with and without medium renewal, we evaluated whether the agitation technique influenced the release profile. Specifically, both agitation methods (magnetic stirring and orbital shaking) yielded similar profile shapes, a trend observed in both the independent batch method and the sample and separate method with medium renewal. The similarity factor (f_2_) and the difference factor (f_1_) were used to compare the different release curves based on their agitation technique, and the results are summarized in Table 1. Each determination was performed up to an endpoint of interest, meeting minimum criteria for similarity evaluation, such as using at least three sampling points (excluding time zero), selecting identical time points for both curves, ensuring no more than one measurement exceeded 85% drug release for either curve, and maintaining a maximum mean difference of 10% between profiles.

For the independent batch method, model-independent analysis revealed that the release profiles obtained with stirring and shaking were not similar, as they exceeded the 10% acceptable difference threshold. For the sample and separate method involving medium renewal, an f_2_ value of 55.9 was obtained, while the f_1_ value was 20.6. These results demonstrate that, under our experimental conditions, the release profiles of RhB from MSNs generated using stirring and shaking agitation techniques are not similar. In the case of the independent batch method, when comparing shaking against stirring generated by a small stir bar, the results showed an f_2_ value of 87.8 and an f_1_ value of 4.6, indicating a high degree of similarity between the release profiles. Similarly, for the method involving replacement of the release medium, the f_2_ and f_1_ values were 91.0 and 2.1, respectively. These data suggest that when a small stir bar is utilized, the release profiles obtained under shaking and stirring agitation conditions can be considered comparable. On the other hand, when the curves obtained using a 20 mm and a 30 mm magnetic stir bar were analyzed (either in the independent batch method or with medium replacement) they did not meet the similarity criteria. For the independent batch method, sampling-point differences exceeded the 10 % limit. Meanwhile, for the method with medium renewal, the f_2_ and f_1_ values were 55.3 and 21.9, respectively, indicating that the curves are not similar. For non-compendial methodologies, careful selection of the stir bar size is essential, as the flow type directly impacts the release process, affecting the reproducibility, robustness, and reliability of the method [46,49,57].

**Figure 4:**
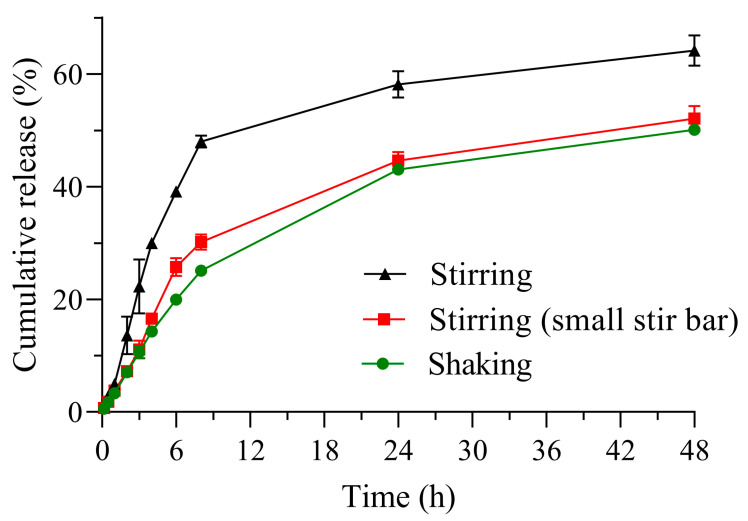
Release profile obtained with the dialysis bag method. Stirring was performed with a 30 mm magnetic bar and the small stir bar was 20 mm long.

The dialysis bag method proposes a different configuration, in which there is a donor compartment inside a membrane and a receiver compartment from which the sample of free drug to be measured is extracted. Although this methodology has been questioned because it does not accurately reflect the true release kinetics from the colloidal system [6,12,58], it remains one of the most popular techniques due to the ease of physically separating the nanoparticle from the released drug before taking a sample from the receiver compartment. When comparing different agitation techniques, the resulting release profiles showed clear differences (Figure 4).

However, profiles obtained with shaking agitation and a small stir bar demonstrated similarity, with an f_2_ value of 76.7 and an f_1_ value of 12.6. This suggests that shaking and stirring with a small bar may be interchangeable under these conditions. In accordance with the above, when comparing release profiles obtained using 20 mm versus 30 mm stir bars, the profiles fell outside the similarity criterion established for the analysis of f_1_ and f_2_. The observed differences between the 30 mm and 20 mm stir bars could be attributed to the higher fluid velocity generated by the larger stir bar, as well as the potential for the dialysis bag itself to contribute to fluid movement within the medium. In contrast, during shaking, the “back-and-forth” motion primarily affects the release medium while having minimal impact on the bag. These results suggest that the increased fluid dynamics induced by the longer stir bar and the movement of the dialysis bag result in greater detection of the dissolved drug in the acceptor compartment.

Furthermore, the variability, expressed as the coefficient of variation (%CV) at each sampling time point, exhibited different trends depending on the agitation technique, which should be considered for future release studies involving nanomedicines. In general, orbital shaking yielded lower %CV values and, consequently, lower variability (Table S1). This suggests that method selection should be carefully considered, particularly for hydrophobic drugs, where variability may be further influenced by drug–medium interactions in addition to the factors described above. Interestingly, at the final experimental time point, regardless of the agitation technique, the percentage of rhodamine B released was similar for both the medium replacement and dialysis methods. Moreover, when employing the 30 mm stir bar, no significant differences in RhB release were observed between the dialysis method and the sample and separate method with medium replacement after 6 h.

#### 3.2.2. Kinetic Analysis of RhB Release

The kinetic data were normalized and fitted to the zero-order, first-order, and power law models to further investigate the release mechanism (Figure S2). The data were analyzed for the entire experimental time range (Table 2). However, for the independent batch method, the release of rhodamine B was analyzed up to 8 h, considering that a plateau was reached at this point. We evaluated the coefficient of determination (R^2^), the Akaike Information Criterion (AIC), and the root mean square error (RMSE) to identify which model provided the best fit to the data (Table S2).

Based on the highest R^2^ and lowest AIC and RMSE values, the RhB release profiles, obtained by sample and separate or dialysis under stirring or shaking, are best described by a first-order model. In the independent batch approach, the average pseudo-first-order rate constants rank as follows: shaking > stirring. In particular, this method yields higher rate constants than both the sample and separate method with medium renewal and the dialysis method. However, this represents an overestimation of the value because, in the independent batch method, the lack of medium renewal slows the process, quickly reaching a plateau and prematurely terminating the release process. For the sample and separate method with medium renewal, the average pseudo-first-order rate constants follow the order: stirring > shaking. For the dialysis bag method, the rate constants also follow the order: stirring > shaking. However, for the sample and separate method with and without medium renewal, the characterization of the release rate expressed through the pseudo-first-order constant is not affected by the agitation technique, yielding consistent values in each case.

Regarding the mechanism, according to the exponential factor n of the power law model, the results suggest a non-Fickian diffusion behavior for the sample and separate method, both with and without medium renewal. This indicates that Fickian diffusion alone does not govern drug release and that surface perturbations caused by the RhB layer may also contribute. In the case of the dialysis bag method, the data suggest a super-Case II transport mechanism, which is most apparent during the initial 8 h. Under these conditions, the release also fits zero-order kinetics, in line with the observed *n* value. However, unlike the sample and separate method—whose kinetics can be described by a pseudo-first-order model—in the dialysis setup the pseudo-first-order behavior actually reflects the RhB permeation through the membrane. Indeed, free rhodamine reaches equilibrium by around 3 h, following first-order kinetics (Figure S3). Beyond this, additional perturbations arise from drug–matrix interactions and the formation of a surface layer of the dye, which also explains why, when fitting only the first 6 h of data, zero-order kinetics appear to apply. Overall, first-order kinetics best describes the complete release process across all methods and agitation techniques evaluated. Nevertheless, the limited agitation inside the dialysis bag exacerbates the effects of surface layering and drug–matrix interactions, which explains the pronounced zero-order fit observed at the initial sampling points. Interestingly, the pseudo-first-order rate constants for the dialysis bag method and the sample and separate method with medium renewal are comparable. This shows that both processes follow first-order kinetics with similar release rates, even though the shapes of their release profiles are different.

Other authors have highlighted the effect of the selection of the sampling method or the agitation technique on the release process. Billings and Anderson compared three in vitro release methods to evaluate gentamicin elution from a collagen matrix [59]. The methods included partial medium replacement and complete medium replacement, both with and without device washing. The results showed significant differences in gentamicin release profiles, with the full medium replacement protocol without washing allowing the highest drug recovery. The authors emphasized that the choice of the sampling method influences both the amount and kinetics of drug release, underscoring the need for standardization in such studies. Recently, Wolska and Szymańska evaluated different in vitro methods to study the release of cyclosporine A and indomethacin from solid lipid particles [26]. They compared three methods: a membrane-free system, a dialysis bag, and a Side-Bi-Side diffusion chamber. The membrane-free system is analogous to the independent batch method used in our study. In this regard, our results align with those reported, as after a certain amount of drug is released, the process slows over time, making it appear as though release has ceased. In our case, we attribute this to the lack of medium renewal and its impact on drug diffusion.

Comparing different methods is essential to properly characterize drug release from nanostructures, as it aids in understanding the physicochemical factors involved, predicting in vivo behavior, and ensuring the biopharmaceutical quality control of formulations. Although these in vitro methods do not replicate in vivo conditions, they can offer useful insights into potential in vivo performance. For instance, differences in release kinetics and agitation sensitivity may reflect physiological barriers such as membrane permeation or fluid dynamics. Therefore, these strategies may help reduce the translational gap by informing early-stage formulation decisions.

## 4. Conclusions

This study systematically compared three non-compendial in vitro methods—sample and separate without medium renewal (independent batch), sample and separate with medium renewal, and dialysis bag—to evaluate the release of a hydrophilic molecule (rhodamine B) from mesoporous silica nanoparticles (MSNs). The results demonstrate that the agitation method (shaking vs. stirring) significantly influences the release profiles, and these techniques should not be interchangeable. The impact of agitation on the release process was evident when assessed using model-independent approaches, but not when analyzed through first-order rate constants. Overall, the findings underscore that both the choice of in vitro method and the agitation technique critically affect the apparent drug release kinetics of drugs from nanocarriers. Among the evaluated methods, the sample and separate approach with medium renewal offers superior consistency and relevance for MSN systems. This study also highlights that parameters such as the vessel-to-agitator ratio, the type of agitation, and the frequency with which the release medium is renewed are essential considerations. These factors should be addressed in future studies aimed at standardized protocols to better bridge in vitro and in vivo performance, supporting early-stage nanoformulation development and regulatory evaluation.

## Figures and Tables

**Table 1:** Calculated values of f_2_ (similarity factor) and f_1_ (difference factor) for RhB release profiles under varied agitation (shaking vs. stirring) and sampling methods. Where values are missing (–), this indicates that the data did not meet the criteria and are considered not similar.

Method		f_2_	f_1_
Independent batch	Shaking vs. Stirring	-	-
	Shaking vs. Stirring (small stir bar)	87.8	4.6
	Stirring vs. Stirring (small stir bar)	-	-
Sample and separate with renewal	Shaking vs. Stirring	55.9	20.6
	Shaking vs. Stirring (small stir bar)	91.0	2.1
	Stirring vs. Stirring (small stir bar)	55.3	21.9
Dialysis bag	Shaking vs. Stirring	-	-
	Shaking vs. Stirring (small stir bar)	76.8	12.6
	Stirring vs. Stirring (small stir bar)	-	-

**Table 2:** Summary of normalized data kinetic parameters up to 8 h for the independent batch method and up to 48 h for the sample and separate method with medium renewal and dialysis bag, for rhodamine B release from MSN.

Method		Zero Order			First Order			Power Law		
		k (% h^−1^) ± SD	R^2^	AIC	k (h^−1^) ± SD	R^2^	AIC	*n* ± SD	R^2^	AIC
Independent batch ^a^	Shaking	17.07 ± 0.23	0.5870	69.8	0.59 ± 0.05	0.9740	45.7	0.83 ± 0.19	0.9904	10.7
	Stirring	14.79 ± 1.81	0.4969	67.5	0.44 ± 0.09	0.9453	46.4	0.52 ± 0.10	0.9777	16.6
	Stirring (small stir bar)	16.11 ± 0.82	0.6457	64.2	0.47 ± 0.06	0.9658	48.2	0.77 ± 0.10	0.9666	21.2
Sample and separate with renewal ^b^	Shaking	2.68 ± 0.02	0.3049	90.3	0.14 ± 0.01	0.9814	53.9	0.64 ± 0.05	0.9931	21.3
	Stirring	2.74 ± 0.05	0.1247	92.9	0.16 ± 0.02	0.9816	52.9	0.65 ± 0.03	0.9929	24.3
	Stirring (small stir bar)	2.71 ± 0.02	0.2459	91.6	0.15 ± 0.01	0.9874	50.5	0.63 ± 0.03	0.9898	23.1
Dialysis bag ^b^	Shaking	2.56 ± 0.02	0.7109	82.5	0.083 ± 0.003	0.9977	32.7	0.94 ± 0.14	0.9923	26.4
	Stirring	2.75 ± 0.01	0.3004	92.6	0.16 ± 0.02	0.9805	55.6	0.83 ± 0.21	0.9633	37.4
	Stirring (small stir bar)	2.61 ± 0.06	0.6174	85.7	0.10 ± 0.01	0.9851	51.3	1.08 ± 0.07	0.9839	35.2

^a^ The analysis was perfomed until 8 h. ^b^ The analysis was perfomed until 48 h.

## Data Availability

The manuscript and supplementary material contain the reported data. Additional relevant data can be obtained upon request from the corresponding author.

## Supplementary Materials

The following supporting information can be downloaded at: https://www.mdpi.com/article/10.3390/pharmaceutics17060694/s1, Table S1. Data set of variability in release profiles, expressed as coefficient of variation (%CV). Table S2. The following table provide the summary of statistical metrics (R^2^, AIC, and RMSE) used to determine the best-fitting kinetic model for the normalized release data of the Figure S3. Figure S1. Release profiles at three agitation speeds (50, 100, and 200 rpm). Profiles obtained using the independent batch (a–c), sample and separate with medium replacement (d–f), and dialysis bag method (g–i). Figure S2. Goodness of fit for the normalized release data. First order model for (a) independent method, (b) sample and separate with medium replacement, and (c) dialysis; zero order model for (d) independent method, (e) sample and separate with medium replacement, (f) dialysis; Power law for g) independent method, (h) sample and separate with medium replacement, (i) dialysis. Figure S3. In vitro release profile of free rhodamine B. The *x*-axis of the main figure has been adjusted to 24 h to show the shape of the graph compared to the profiles of RhB released from nanomaterials. The inset figure shows the release with the *x*-axis adjusted to 3 h. Equation (S1). The Reynolds number was obtained from the following Equation (S1).
