# Supplementary material for: Kinetic and Methodological Insights into Hydrophilic Drug Release from Mesoporous Silica Nanocarriers
Source: Pharmaceutics. 2025 May 25;17(6):694. doi: 10.3390/pharmaceutics17060694 (PMC12195950; doi:10.3390/pharmaceutics17060694)
Supplement: Supplementary file 1 [file pharmaceutics-17-00694-s001.zip › pharmaceutics-3652397-supplementary.pdf]

Supplementary materials

Kinetic and methodological insights into hydrophilic drug release from mesoporous silica nanocarriers

Rodrigo Rozas[1,2], Andrea C. Ortiz[3], Sofía Peñaloza[1], Sebastián Lizama[2], Mario E. Flores[4], Javier Morales[1,*] and Francisco Arriagada[1,*]

[1] Departamento de Ciencias y Tecnología Farmacéuticas, Facultad de Ciencias Químicas y Farmacéuticas, Universidad de Chile, Santiago 8380494, Chile

[2] Instituto de Farmacia, Facultad de Ciencias, Universidad Austral de Chile, Valdivia 5090000, Chile

[3] Escuela de Química y Farmacia, Facultad de Ciencias, Universidad San Sebastián, Santiago 7510157, Chile

[4] Instituto de Ciencias Químicas, Facultad de Ciencias, Universidad Austral de Chile, Valdivia 5090000, Chile

*Corresponding Authors:

Dr. Francisco Arriagada

E-mail address: francisco.arriagada@ciq.uchile.cl

Dr. Javier Morales

E-mail addres: javiermv@ciq.uchile.cl

Table S1. Data set of variability in release profiles, expressed as coefficient of variation (%CV).

Time	Sample and separate						Dialysis		
	Independent batch			Medium replacement					
	%CV small stir bar	%CV stirring	%CV shaking	%CV small stir bar	%CV stirring	%CV shaking	%CV small stir bar	%CV stirring	%CV shaking
0.15	7.6	10.5	2.7	4.4	9.7	1.7	33.1	33.0	18.9
0.5	8.3	8.2	5.1	1.7	7.3	1.4	3.4	16.1	17.1
1	4.7	11.1	2.8	0.9	5.4	3.8	5.9	5.0	10.2
2	2.6	6.6	3.3	1.1	6.2	1.6	3.2	24.7	10.4
3	1.4	5.2	2.8	1.1	3.9	1.0	14.3	21.6	9.8
4	4.9	6.0	1.2	2.9	2.9	2.6	5.8	2.7	6.0
6	2.2	5.1	3.5	2.6	3.0	1.1	6.2	1.7	5.0
8	3.3	4.3	1.7	6.1	5.8	0.8	4.5	2.3	2.8
24	0.8	1.6	1.9	3.4	1.3	1.2	3.4	3.9	1.3
48	-	-	-	4.3	2.1	0.5	4.3	4.2	1.9

Table S2. The following table provide the summary of statistical metrics (R^2, AIC, and RMSE) used to determine the best-fitting kinetic model for the normalized release data of the Figure S2.

		**Criteria**								
**Method**		Zero order			First order			Power law		
		R^2	AIC	RMSE	R^2	AIC	RMSE	R^2	AIC	RMSE
**Independent batch**[a]	Shaking	0.5870	69.8	25.05	0.9740	45.7	6.08	0.9904	10.7	11.77
	Stirring	0.4969	67.5	22.78	0.9453	46.4	7.54	0.9777	16.6	8.77
	Stirring (small stir bar)	0.6457	64.2	21.70	0.9658	48.2	6.70	0.9666	21.2	10.08
**Sample and separate with renewal**[b]	Shaking	0.3049	90.3	26.65	0.9814	53.9	3.65	0.9931	21.3	6.00
	Stirring	0.1247	92.9	30.44	0.9816	52.9	4.29	0.9929	24.3	7.74
	Stirring (small stir bar)	0.2459	91.6	28.44	0.9874	50.5	3.19	0.9898	23.1	7.85
**Dialysis bag**[b]	Shaking	0.7109	82.5	18.07	0.9977	32.7	1.51	0.9923	26.4	6.57
	Stirring	0.3004	92.6	29.83	0.9805	55.6	4.51	0.9633	37.4	11.88
	Stirring (small stir bar)	0.6174	85.7	21.25	0.9851	51.3	3.84	0.9839	35.2	8.64

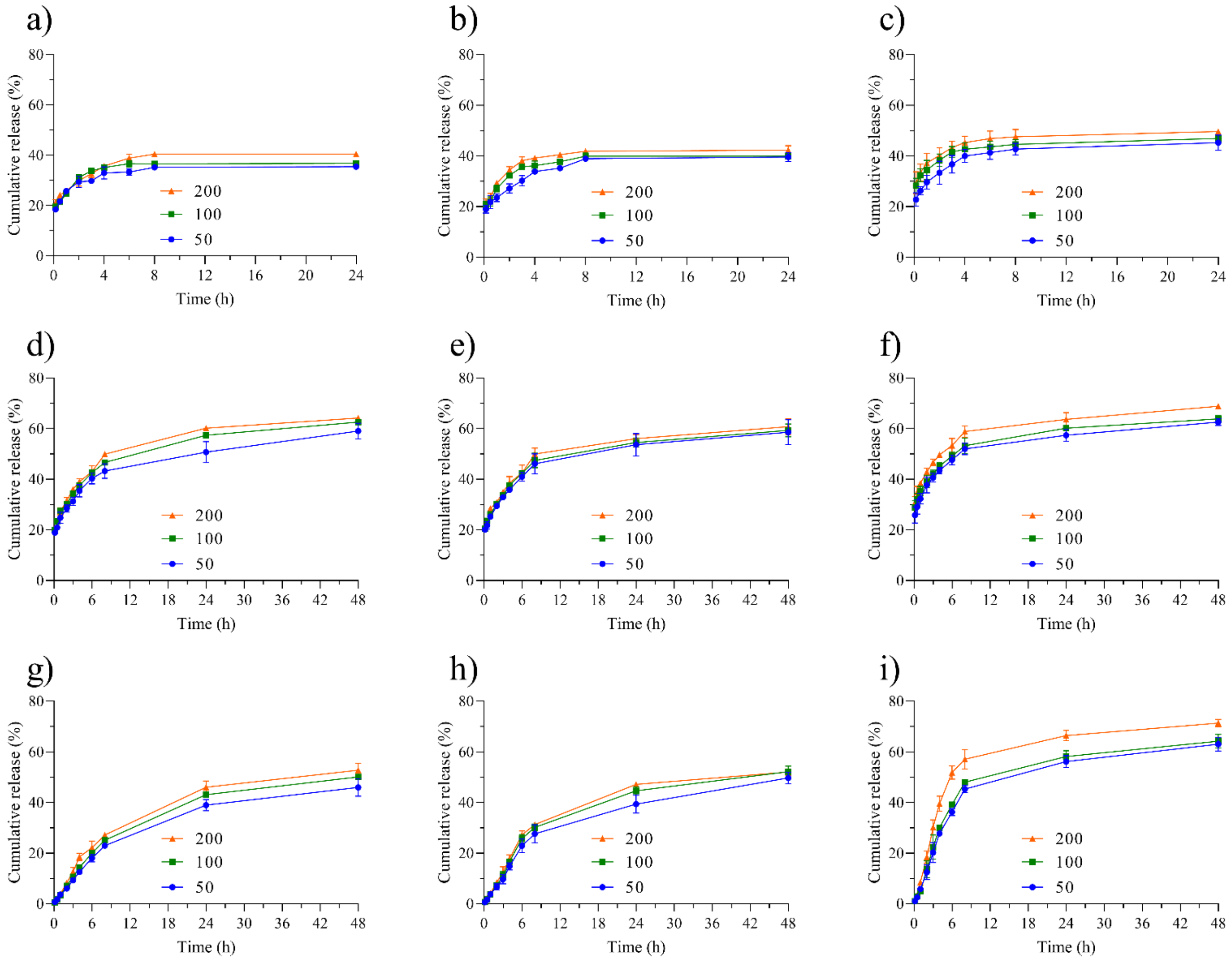

Figure S1. Release profiles at three agitation speeds (50, 100, and 200 rpm). Profiles obtained using the independent batch (a-c), sample and separate with medium replacement (d-f), and dialysis bag method (g-i).

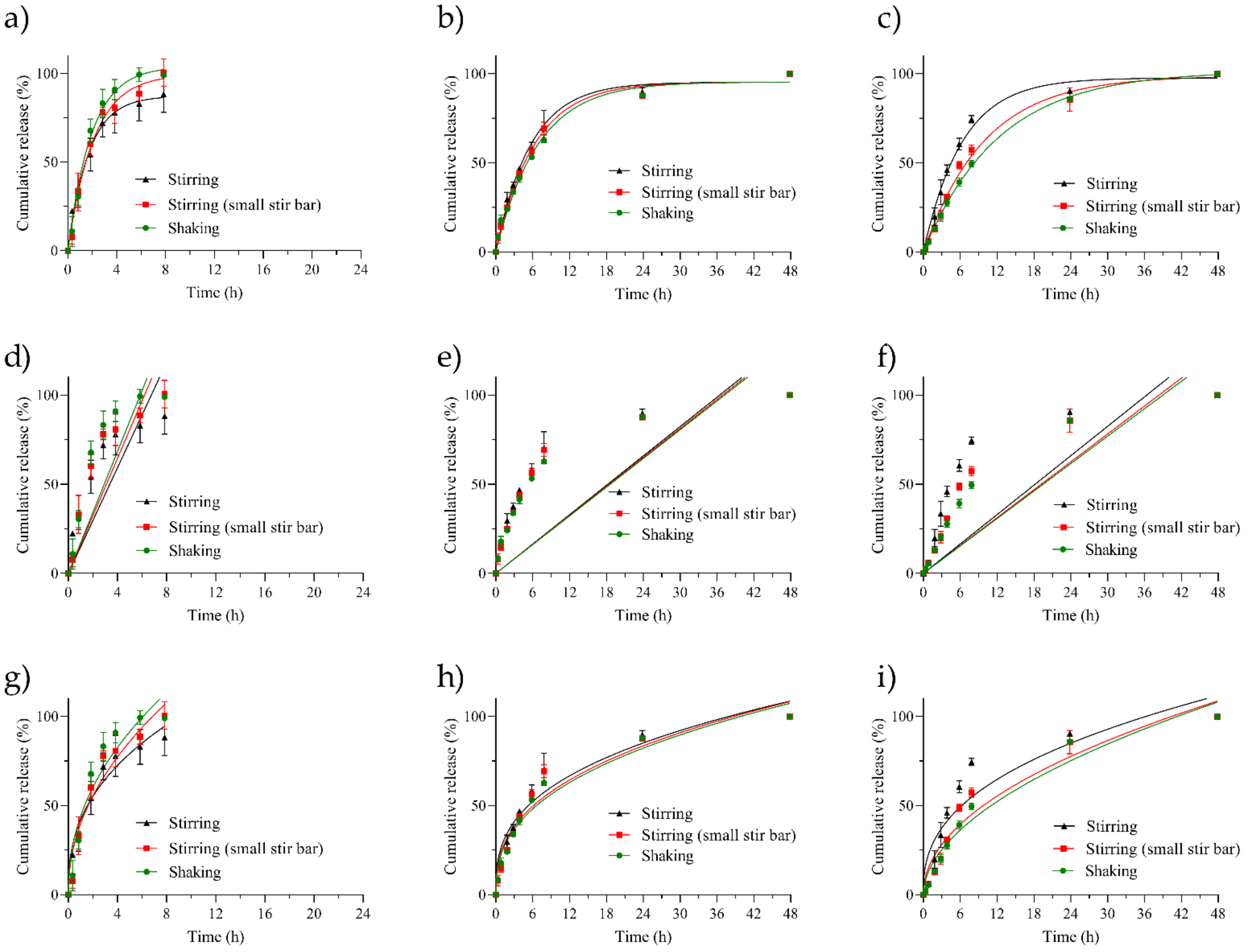

Figure S2. Goodness of fit for the normalized release data. First order model for a) independent method, b) sample and separate with medium replacement, and c) dialysis; zero order model for d) independent method, e) sample and separate with medium replacement, f) dialysis; Power law for g) independent method, h) sample and separate with medium replacement, i) dialysis.

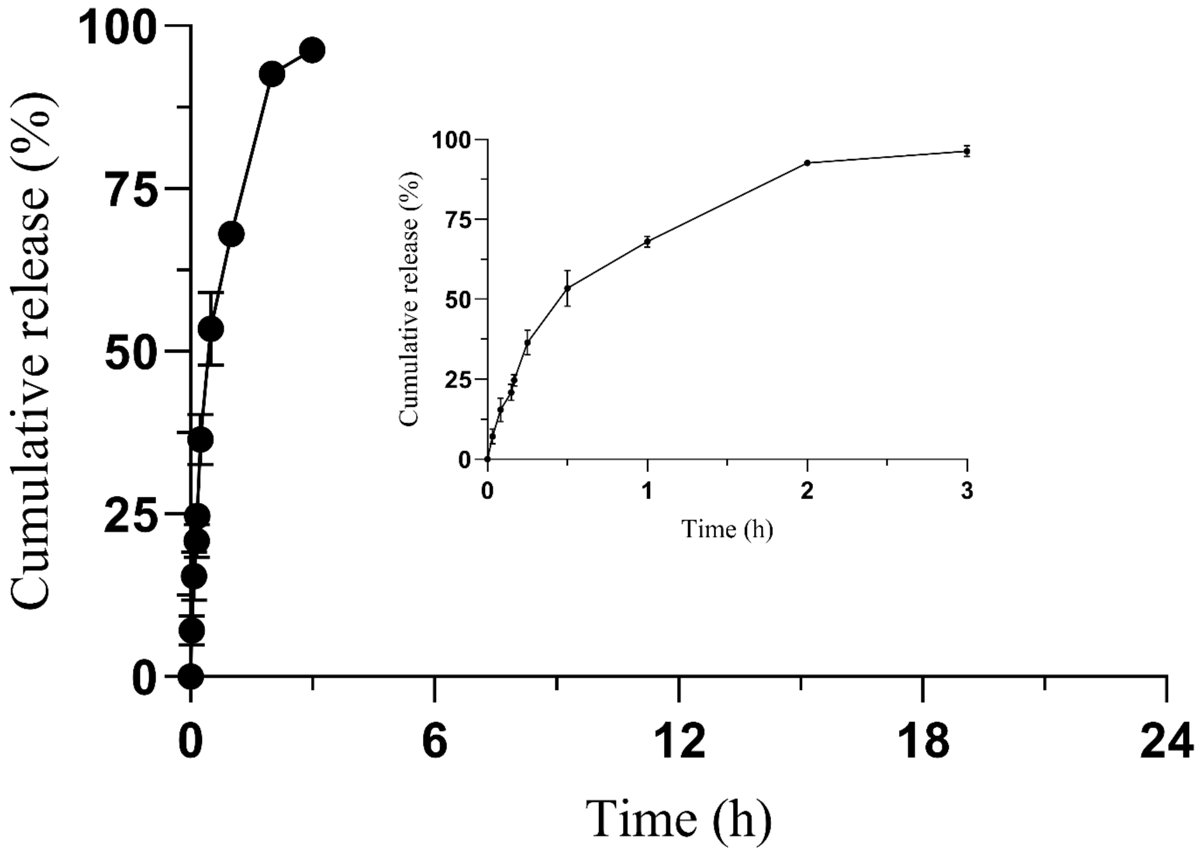

Figure S3. In vitro release profile of free rhodamine B. The x-axis of the main figure has been adjusted to 24 h to show the shape of the graph compared to the profiles of RhB released from nanomaterials. The inset figure shows the release with the x-axis adjusted to 3 h.

The Reynolds number was obtained from the following equation S1:

$$R_e = \frac{N{D_{stir\ bar}}^2}{\nu} \quad \text{(S1)}$$

Where ν is the kinematic viscosity of the fluid (6.965×10^{-7} m^2/s), N is the rotation speed of the bar (100/60 rotation/s) and $D_{stir\ bar}$ is the length of the bar (0.02 or 0.03 m).
